# The Interactions of microRNA and Epigenetic Modifications in Prostate Cancer

**DOI:** 10.3390/cancers5030998

**Published:** 2013-08-09

**Authors:** Prashant Kumar Singh, Moray J. Campbell

**Affiliations:** Department of Pharmacology & Therapeutics, Roswell Park Cancer Institute, Elm and Carlton Streets, Buffalo, NY 14263, USA

**Keywords:** epigenetics, DNA methylation, histone modifications, microRNA, prostate cancer, cancer

## Abstract

Epigenetic modifiers play important roles in fine-tuning the cellular transcriptome. Any imbalance in these processes may lead to abnormal transcriptional activity and thus result in disease state. Distortions of the epigenome have been reported in cancer initiation and progression. DNA methylation and histone modifications are principle components of this epigenome, but more recently it has become clear that microRNAs (miRNAs) are another major component of the epigenome. Interactions of these components are apparent in prostate cancer (CaP), which is the most common non-cutaneous cancer and second leading cause of death from cancer in the USA. Changes in DNA methylation, altered histone modifications and miRNA expression are functionally associated with CaP initiation and progression. Various aspects of the epigenome have also been investigated as biomarkers for different stages of CaP detection, though with limited success. This review aims to summarize key aspects of these mechanistic interactions within the epigenome and to highlight their translational potential as functional biomarkers. To this end, exploration of TCGA prostate cancer data revealed that expression of key CaP miRNAs inversely associate with DNA methylation. Given the importance and prevalence of these epigenetic events in CaP biology it is timely to understand further how different epigenetic components interact and influence each other.

## 1. Epigenetic Contributions to Cancer

### 1.1. An Epigenetic Basis for Prostate Cancer

Prostate cancer (CaP) is most common noncutaneous cancer diagnosed and second leading cause of death in men from cancer in the United States [1,2]. This cancer is highly heterogeneous, ranging from asymptomatic to a rapidly fatal systemic malignancy. Several genetic and epigenetic alterations are highly prevalent and appear to be important factors in the tumorigenesis and progression of CaP. Most common genetic changes associated with CaP includes deletions involving NK3 homeobox 1 (*NKX3.1*) [3] and phosphatase and tensin homologue tumor suppressor genes (*PTEN*) [4,5], amplifications of the androgen receptor (*AR*) [6] and *MYC* [7] genes and more recently described translocations that lead to *TMPRSS2:ERG* and *SLC45A3:ERG* gene fusions [8,9]. Recently, tumor analyses with next generation sequencing approaches have identified several additional novel somatic mutations, including *MED12*, *FOXA1* and *SPOP*. The E3 ubiquitin ligase adaptor speckle-type poxvirus and zinc finger (POZ) domain protein (*SPOP*) showed recurrent mutations in 6–15% of tumors across multiple independent cohorts [10].

The complex nature of cancer phenotypes however cannot be explained by genetic components alone [11]. Epigenetic modifications appear to contribute significantly to transformation. These events can be defined as heritable changes to the expression and regulation of gene expression that are not associated with alteration in the DNA sequences [12]. These epigenetic events including DNA methylation and histone modifications appear to play distinct yet complementary roles to genetic events and add to a fuller explanation for the basis of cancer. For example, many cancers show gene-specific and global changes in DNA CpG methylation and/or altered histone modification patterns [13,14].

Among the epigenetic modifications, altered DNA methylation and histone modification patterns have been frequently reported in CaP [15,16,17]. These epigenetic modifications govern tissues specific gene regulation by a complex machinery of several DNA and histone modifying enzymes including DNA methyltransferases (DNMTs) [18], histone acetyltransferases (HATs) [19], histone deacetylases (HDACs) [20], histone methyltransferases (HMTs) [21], histone demethylases (HDMTs) [22], and chromatin remodeling enzymes (reviewed in [13]).

One of the most prominent epigenetic modifications reported in CaP is hypermethylation of glutathione S-transferase (*GSTP1*) gene. *GSTP1* is involved in detoxification of genotoxic and electrophilic compounds. Hypermethylation of *GSTP1* is reported in 70 to 90% of patients with CaP but not in benign hyperplastic prostate tissue [23]. Similarly, up-regulation of HMT enhancer of zeste homolog 2 (*EZH2*) is reported in metastatic CaP, and localized CaP with higher expression of *EZH2* show poorer prognosis [24]. Also, high expression of *EZH2* has been reported in hormone-refractory CaP compared to benign hyperplastic prostate tissue [24,25]. EZH2 catalyzes the trimethylation of histone H3 on Lys 27 (H3K27) and is involved in gene repression and contribute to CaP tumorigenesis through silencing of tumor suppressor genes.

Recently published ENCODE project data has greatly enhanced the understanding of transcriptional landscape of the genome. Analysis across multiple cell lines suggested that as much as 75% of the genome is transcribed into different types of RNA molecules e.g., protein coding, long non-coding, pseudogenes and small RNA genes [26]. Although this percentage is the subject of significant debate [27,28], it is clear that the rate, magnitude and diversity of RNA transcription is greater than previously suspected. Of these RNA species, the microRNAs (miRNAs) have emerged as major biological regulators. MiRNAs are small (about 19–22 nucleotides) non-coding RNA molecules and modulate the expression of many, if not all, genes in the human genome. They act by inhibition of protein translation and/or degradation of mRNA transcripts [29]. Understanding their function has contributed significantly to the knowledge of how genomic information is interpreted in physiology and corrupted in malignancy [30].

It is important to understand the interplay of DNA methylation, histone modifications and miRNA and their cumulative effect on CaP initiation and progression. In last five years considerable work has been undertaken and has begun to reveal the true extent of epigenetic dis-regulation of the genome in cancer. In this review we particularly focus on how miRNAs and DNA/histone modifications interact and regulate each other and how they can be used in CaP diagnosis and treatment.

### 1.2. An Emergent Understanding on the Role of miRNA in Prostate Cancer

In parallel to the well-established understanding on the role of classical epigenetic events, the role of non-coding miRNA in epigenetic regulation of gene expression has also emerged. The four types of small RNA genes, namely, small nuclear (sn)RNAs, small nucleolar (sno)RNAs, miRNAs and transfer (t)RNAs correspond to 85% of total small RNAs (7,053) annotated by GENCODE. Importantly, ENCODE data revealed the wide range expression of miRNAs in different normal and cancer cell lines [26]. At any given time point 28% of miRNAs (497 of 1,756) were expressed in at least one cell line. Interestingly 30% (147 of 497) of these miRNAs were detected in all 12 cell lines analyzed [26]. This observation strengthens the idea that miRNAs are important components of gene regulation networks and expressed differentially in different disease models. Furthermore, de-regulation of miRNAs has been shown for a variety of solid tumors, including breast, colon, and prostate [31,32,33]. Thus, these studies have established an important role of miRNAs in the control of gene regulation and they are frequently de-regulated in many cancers [34,35].

Although miRNAs represent only ~1% of the genome but they are estimated to target 30% of genes [36]. The relatively small number of miRNAs and their wide coverage of protein coding genes make them very attractive to be used as disease biomarkers. Compared to mRNA signatures miRNAs have better and strong biomarker properties [37]. It has been estimated that miRNAs provide 20 times more power in biomarker studies as compared to mRNAs (when comparing 20,000 mRNAs to ~1,000 miRNAs), which makes them ideal candidates for studies with small sample size [37].

In CaP, several miRNAs have been reported to be differentially regulated and act as both tumor suppressor and onco-miRs. For instance, miR-221 and miR-222 have been shown to be upregulated in castration-resistant prostate cancer (CRPC) cells compared to androgen-dependent CaP cell line and significantly affect the response to DHT [38]. In general, oncogenic properties of miR-221 and miR-222 are attributed to their control of the cyclin dependent kinase (Cdk) inhibitors p27^KIP1^ and p57^KIP2^, and thus control of G_1_ to S phase transition [39,40], PI3K and PTEN signaling [41] and other targets implicated in malignant transformation, including *CX43* [42], *RECK* [43], and *ERα* [44,45].

Lin *et al*. observed that aggressive tumors (Gleason grade ≥7) had elevated expression of miR-221 and miR-222 compared to less aggressive tumor tissues (Gleason grade <7), further suggesting an oncogenic role of these miRNAs in CaP development [46]. Conversely, others have shown the loss of miR221/222 in early stage [47] as well as in aggressive CaP [48,49]. Most recently, a miRnome wide scan also found miR-221 and miR-222 to be downregulated when comparing 20 matched pairs of microdissected tissue samples of prostate cancer and non-tumor tissue [50]. These conflicting results perhaps indicate that expression levels are differentially modulated at divergent disease stages.

Similarly, miR-125b has been identified in CaP along with miR-143 to be upregulated in metastatic CaP serum samples as compared to normal individuals [51]. Expression of miR-125b in serum of CaP patients is reported to be upregulated as compared to normal controls [51] whereas other studies reported it to be downregulated in CaP as compared to normal or benign prostatic hyperplastic (BPH) samples [47,52,53]. MiR-125 regulates cell proliferation in prostate cancer cell lines [54], and it has been suggested to be upregulated by androgen signaling [56]. Functionally in CaP, miR-125b has been reported to target *BAK1* [55] (a pro-apoptotic member of the BCL-2 gene family) and *EIF4EBP1* (Eukaryotic translation initiation factor 4E-binding protein 1), a gene that encodes one member of a family of translation repressor proteins [52].

Other important miRNAs involved in CaP includes miR-21 which is an AR-regulated miRNA and overexpressed in CRPC compared to adjacent normal tissue [56]. Overexpression of miR-21 was also observed in docetaxel resistant variant of PC3 cells, additionally, ectopic expression of miR-21 in wild type PC3 cells increased the resistance to docetaxel [57]. The oncogenic and drug resistance properties of miR-21 were attributed to its control of downstream target, programmed cell death 4 (*PDCD4*) [57,58].

Several miRNAs have also been identified as tumor suppressors in Cap including miR-143, miR-145 and miR-200 family. Expression of miR-143 and miR-145 are significantly suppressed in CaP and negatively associate with metastasis [59]. These miRs contribute to CaP progression through epithelial-mesenchymal transition (EMT) [59] and loss of their repressive effect on EGFR/RAS/MAPK pathway [60]. MiR-205 and miR-200 family miRNAs are also downregulated in CaP and have been shown to regulate EMT by targeting *ZEB1* and *ZEB2* in CaP [43,60,61].

## 2. Interplay between Histone Modifications, DNA Methylation and miRNA

Critically, the three different epigenetic modifications *i.e.*, DNA methylation, histone modifications and miRNAs interact and influence each other [62]. For example, expression of miRNAs can be regulated by DNA methylation in their respective promoter regions [63]. Similarly miRNAs can directly target chromatin remodeling enzymes [64] and DNA methyltransferases (DNMTs) [65] and in turn modulate the downstream effects.

### 2.1. Regulation of DNA and Histone Modifications under the Control of miRNAs in Prostate Cancer

MiRNAs control gene expression post-transcriptionally by translational inhibition when base pairing between miRNA and target sequence is imperfect, while perfect or near perfect complementarity can induce the degradation of the target mRNA [66]. One miRNA can target several genes and one gene can similarly be targeted by several miRNAs [66]. The latest release of miRBase (miRBase 19) has 1,595 primary transcripts and 2,038 mature miRNA sequences. The wide range of miRNA targets makes them essential in the majority of biological processes, including cell cycle regulation, differentiation, development, metabolism and aging.

Although the biogenesis of miRNA has been well described, the regulation of miRNA expression remains less well explored. Several miRNAs have well-defined promoters but for many the regulatory elements are not as clear. They are often present in introns of protein coding genes and the promoters for the genes are considered to be promoters for the intragenic miRNAs [66,67]. Interestingly, there is evidence for differential regulation between the host mRNA and miRNA and may suggest an emergent role for alternative regulation processes.

**Figure 1:**
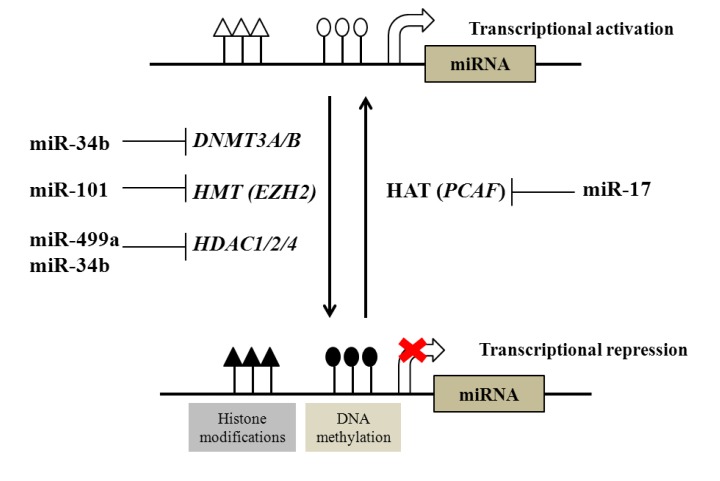
Interactions of DNA/histone modification and miRNA. Epigenetic regulatory mechanisms such as DNA methylation (controlled by DNMTs) and histone modifications modulate epigenetic states and transcriptional responsiveness of both mRNA and miRNA target genes. These actions are controlled by antagonistic classes of enzymes, for example by histone deacetylases (HDACs) and histone acetyl transfereases (HATs). There is good evidence for control of their expression by miRNA. For example, miR-101 targets include EZH2 [71] and miR-499a targets include HDAC1 [72], whereas the HAT (p300/CBP-associated factor (PCAF)) is targeted by miR-17-5p [73].

Like their target genes, miRNAs can also directly or indirectly target key effectors of the epigenetic machinery (Figure 1). For example, miR-29 family members (29a, 29b, and 29c) are downregulated in several cancers and they are predicted to target DNA methyltransferase *DNMT3A*, *DNMT3B* and *DNMT1*. Indeed, enforced expression of miR-29 family members resulted in the loss of DNA methylation in lung cancer and acute myeloid leukemia and re-expression of methylation silenced genes [68,69]. Recently, miR-34b has been shown to target *DNMT1*, *HDAC1*, *HDAC2* and *HDAC4* in CaP cell lines [70]. Interestingly, miR-34b was also epigenetically silenced by DNA methylation and ectopic expression of miR-34b in CaP cells resulted in partial demethylation of 5' upstream sequence of the miR-34b gene and also showed enrichment of trimethylated histone H3 lysine 4 (H3K4me3), mark for an active chromatin [70]. These data provide an insight into interplay between miRNA and DNA/histone marks in CaP.

A similar miRNA-mediated epigenetic cross-talk occurs with key histone modifications. Polycomb proteins form chromatin-modifying complexes that induces gene silencing by catalyzing tri-methylation of histone H3K27. There are two polycomb repressor complexes (PRCs), PRC1 and PRC2 and deregulation of these complexes has been reported to associate with many cancers [74,75]. For example, *EZH2*, is a highly conserved catalytic subunit within PRC2, and has been reported to be elevated in CaP and correlate with metastatic CaP and poor prognosis in localized CaP [17]. One of the important mechanisms for altered PRC2 activity arises due to elevated *EZH2* level that occurs through down-regulation of miR-101 [76]; expression of miR-101 is frequently lost in CaP [34,35]. Increased *EZH2* expression is associated with CaP progression and loss of miR-101 may be responsible for increased *EZH2* expression in CaP [71]. MiR-101 also plays important roles in control of cell proliferation, and its expression correlates inversely with aggressiveness in CaP cell lines models [71]. Re-expression of miR-101 inhibits the expression of *EZH2* and attenuates cell proliferation and tumor invasiveness [71]. More recently, knock-down of Dicer, a key protein required for miRNA processing, resulted in increased expression of other PRC components e.g., *EZH2*, *EED*, *& SUZ12* (PRC2 components) and *BMI1* and *RING2* (PRC1 components). Together these data suggest miRNA control of the PRC complex occurs through multiple targets [77].

Upon H3K27 methylation by PRC2 proteins, PRC1 proteins are recruited to chromatin to maintain stable gene silencing by catalyzing ubiquitinylation of histone H2A. *BMI1* is negatively regulated by miR-203, 200b/c while *RING2* is negatively regulated by miR-181a/b and miR-200b/c [77]. Downregulation of *BMI1* and *RING2* by these miRNAs also resulted in decreased global ubiquityl-H2A. Interestingly, expression of these miRNAs was in turn regulated by *EZH2* and silencing of *EZH2* or expression of miR-101 resulted in overexpression of these miRs, and reflects the intricate co-regulation of miRNA and mRNA in feed forward loop structures [67,77,78,79,80,81,82,83]. These finding illustrated the complex interplay of epigenetic machinery and miRNA in controlling gene silencing in CaP.

MiRNA also regulate the expression of histone deacetylases (HDACs) that control gene silencing by catalyzing deacetylation of key histone residues. The class I HDACs are the most frequently overexpressed in cancers, particularly *HDAC1*, which is found to be overexpressed in CaP [84]. *HDAC1* is a direct target of miR-499a in PC-3 cells [72], and in turn miR-449a is involved in cell-cycle regulation and is frequently downregulated in CaP. Overexpression of miR-449a in CaP cell lines leads to downregulation of *HDAC1* and cell cycle arrest. The locus specific *versus* global effects of HDACs are emerging and interestingly the growth arrest induced by miR-449a mediates repression of *HDAC1* leading to the induction of *p27^(kip1)^* [72], again suggesting the combinatorial epigenetic regulation of key growth regulators.

Another class of important histone modifiers are the histone acetyltransferases (HATs). They acetylate core histones, which is associated with easily accessible chromatin for the transcriptional machinery. In CaP p300/CBP-associated factor (PCAF), which has HAT activity, has been shown to act as a co-activator for androgen receptor (AR) [85]. Gong *et al*. showed that PCAF was upregulated in CaP cell lines compared to normal prostate epithelium cells and promotes DHT-stimulated AR transcriptional activity. Interestingly, they also showed that *PCAF* is a direct target of miR-17-5p, and it attenuated DHT-induced expression of the PSA gene and inhibited DHT-induced cell growth in LNCaP cells [73]. Taken together these observations suggest that miRNAs play an important role in fine tuning of both silencing and activating epigenetic marks in CaP (Table 1).

**Table 1:** Interaction of miRNA and epigenetic modifications.

Examples of miRNAs regulated by DNA methylation			
DNA methylation	miR-145, miR-193b, miR-34a, miR-34b, miR-200c, miR-141, miR-205, miR-196b, miR-21, miR-615		
**Examples of epigenetic modifiers regulated by miRNAs**			
**Epigenetic modifiers**			**Targeting miRNA**
DNA methyltransferases (DNTMTs)	*DNMT3A* and *DNMT3B*		miR-29 family
	*DNMT1*		miR-34b
Histone methyltransferases (HMTs)	*EZH2*		miR-101
Histone deacetylases (HDACs)	*HDAC1*, *HDAC2 and HDAC4*		miR-34b, miR-499a
Histone acetyltransferases (HATs)	*PCAF*		miR-17-5p

### 2.2. Regulation of microRNAs by DNA and Histone Modification in Prostate Cancer

Genes encoding miRNAs are as tightly regulated as any other gene in the genome and thus miRNA expression is also subjected to similar epigenetic regulation (Figure 1). Many miRNAs are present in introns of protein coding genes and they are regulated by the promoter of host gene. In this manner the epigenetic regulation of protein-coding genes can also affect the expression of miRNA. Also, many miRNAs are located in clusters in the genome and therefore more than one miRNA can be regulated in parallel by a single transcriptional event. In the genome, distribution of CpG islands around miRNA genes is similar to protein coding genes [86]. For example, mapping to the genome of differentially regulated miRNAs after DNA methyltransferase inhibition with 5-azacytidine (5-aza) showed that between 13 and 24% of human miRNA genes are located within 3 and 10 kb of a CpG island, respectively [86].

Several miRNAs are frequently deregulated in different cancers and many of them are epigenetically regulated. In CaP, miR-200 family members and miR-205 specifically are frequently altered and regulate EMT and cell migration/invasion [60,87]. Members of the miR-200 family include five miRNAs transcribed from two clusters, one on chromosome 1 encoding miR-200b, miR-200a, and miR-429 and other on chromosome 12 encoding miR-200c and miR-141. Members of miR-200 family combined with miR-205 regulate EMT and cell migration/invasion by directly targeting *ZEB1* and *ZEB2* [61]. MiR-205 is downregulated in the majority of cancers including CaP [88]. One of the proposed mechanisms for downregulation of miR-205 is through methylation of its promoter, and has been shown to be associated with chemotherapy drug resistance [89]. Specifically, downregulation of miR-205 arises due to a hypermethylated promoter, and the expression of miR-205 increased when cells were treatment with 5-aza. Similarly, miR-200c and miR-141 are downregulated in PC-3 cells as compared to LNCaP, and showed hypermethylation of the promoter CpG region in PC-3 and unmethylated promoter CpG in LNCaP cells. Indeed treatment with 5-aza increased the expression of miR-200c and miR-141 in PC-3 cells [90]. These findings suggest repression of miR-200c/miR-141 is epigenetically controlled in CaP.

Another miRNA that is frequently downregulated in cancers is miR-34a [91,92]. The expression of miR-34a is regulated by p53, and perhaps reflecting this, it induces apoptosis and cell-cycle arrest [93,94]. Again, miR-34a is downregulated in many CaP cell lines and the promoter region of miR-34a is hypermethylated in malignant CaP cell lines as compared to non-malignant or benign prostate hyperplasia [92]. Interestingly, similar to hypermethylation observed in CaP cell lines, the promoter of miR-34a has also been demonstrated to be methylated in the majority (79%) of primary CaP tumors as compared to normal prostate stroma [92]. Additionally, miR-34a is inactivated by aberrant CpG methylation in many other types of cancer cell lines including lung (29.1%), breast (25%), colon (13%), kidney (21.4%), bladder (33.3%) and pancreatic carcinoma (15.7%) [92]. Intriguingly, miR-34a has been shown to regulate cancer stem cell (CSCs) properties in CaP and downregulation of miR-34a in prostate cancer stem cells may contribute to metastasis by regulating migratory, invasive and metastatic properties of CSCs [95].

Other workers have investigated repressed miRNA in CaP. For example, Rauhala *et al*. searched for miRNAs silenced by DNA methylation in CaP cell lines and identified miR-193b to be silenced in 22Rv1 and VCaP cell lines. Expression of miR-193b was increased after treatment with 5-aza and trichostatin A (TSA) [96]. Also, miR-193b expression was significantly reduced in CaP tumors compared to adjacent normal tissue. Methylation levels at a CpG island located ~1 kb upstream of the mature miR-193b locus were higher in CaP cell lines compared to a normal prostate epithelial cell line. The methylation was partially removed after treatment with 5aza in combination with TSA, and expression of miR-193b was restored in 22Rv1 cells [96]. Overexpression of miR-193b caused reduction in cell growth and an inhibition of anchorage independent growth suggesting that miR-193b is an epigenetically silenced putative tumor suppressor in CaP [96].

Similarly, miR-145 has been reported as tumor suppressor and is frequently downregulated in many cancers including CaP [97]. MiR-145 is also regulated by p53 and inhibits tumor cell growth and invasion by targeting several genes such as c-MYC [98,99]. In CaP, miR-145 is down regulated in cell lines as well as 81% of primary tumors compared to adjacent normal tissue [100,101]. The promoter of miR-145 was hypermethylated in CaP cell lines and proximal CpG sites were completely methylated in all three cell lines tested (PC3, LNCaP and DU145), whereas distal CpG’s showed complete methylation in PC3 and partial methylation in other two cell lines [100]. The miR-145 promoter was most methylated in PC3 cells and treatment with 5-aza increased the expression of miR-145 in all three cell lines [100]. Another study from the same group revealed that the promoter of miR-145 was methylated in CaP tumors and the expression of miR-145 also correlated with mutation status of p53 [101]. Of all the CaP tumor samples tested, 81% showed downregulation of miR-145 of which 35% had both methylation of miR-145 promoter and p53 mutation, 29% had p53 mutation only and 18% had methylation only. Interestingly, four CaP samples with no change in miR-145 expression had neither p53 mutation nor hypermethylation of miR-145 and 9 of 10 BPH samples with high expression of miR-145 showed no hypermethylation of miR-145 [101]. They also showed that methylation of miR-145 promoter inhibits the binding of p53 and regulation of miR-145 by p53. Renewal of miR-145 promoter activity and p53 binding led to increased apoptosis [101].

Whilst these candidate approaches are illustrative of concepts, genome-wide approaches are required to reveal the full significance of interaction between miRNA and epigenetic modifiers. Hulf and co-workers performed integrative analysis combining primary transcription, genome-wide DNA methylation and H3K9Ac marks with miRNA expression to identify epigenetically regulated miRNA in cancer [102]. They identified three epigenetically silenced miRNAs and one activated miRNA in CaP by using the normal prostate epithelial (PrEC) and LNCaP cells with three criterion; primary transcript expression, DNA methylation loss and H3K9Ac gain, and identified miR-205, miR-196b and miR-21 to be epigenetically repressed and miR-615 to be epigenetically activated. As discussed above, miR-205 has been shown to be epigenetically silenced in CaP [89]. Also, miR-21 has previously been shown to be epigenetically regulated in ovarian cancer [103], and may be similarly regulated in CaP. The role of miR-196b and miR-615 is not very clear in CaP but it is interesting to note that in LNCaP cells, the HOXC cluster on chromosome 13, where miR-615 is transcribed from, is epigenetically regulated [104].

## 3. Clinical Exploitation of Epigenetic States in Prostate Cancer

### 3.1. Epigenetic Modifications as Biomarkers in Prostate Cancer

Although, the commonly used prostate-specific antigen (PSA) test has significantly increased the detection of clinically localized tumors, there are many concerns regarding sensitivity and specificity of the PSA test [105]. Therefore, there is an urgent need to identify either more accurate biomarkers for CaP or to establish markers that can be used in combination with PSA for more accurate CaP diagnosis. Understanding the molecular changes during CaP development and progression may help identify accurate biomarkers.

Accumulating evidence for the role of epigenetic modifications and miRNAs during CaP development suggests that they can be used in more accurate diagnosis of CaP initiation and progression and in turn be translated as a functional biomarker. DNA methylation has been frequently studied in CaP and contributes to both the disease initiation and progression [106,107]. As discussed previously, methylation of *GSTP1* promoter is most frequent epigenetic modification in CaP. Hypermethylation of *GSTP1* promoter is present in 90% of adenocarcinomas and 70% of high-grade prostatic intraepithelial neoplasia (high-grade PIN) lesions but not in normal prostate epithelium or hyperplastic epithelium [23]. Analysis of methylation in tumor samples is highly invasive, which is not an ideal biomarker property. However, *GSTP1* methylation can be detected in body fluids, *i.e.*, urine and serum with high specificity (86.8–100%) but low sensitivity in both urine (18.8–38.9%) and serum/plasma (13.0–72.5%) [108,109,110]. Hence, analysis of multiple gene methylation patterns along with *GSTP1* may provide increased specificity. Hoque *et al*. used promoter methylation patterns of nine genes in urine sediment DNA to distinguish normal and CaP individuals [108]. In general, methylation patterns in urine samples showed correlation with the methylation status in the primary tumor. A combination of only four genes (*p16*, *ARF*, *MGMT*, and *GSTP1*) was able to detect 87% of prostate cancers with 100% specificity [108]. Similarly, Rouprêt *et al*. showed that the promoter methylation pattern of four genes, *GSTP1*, *RASSF1a*, *RARbeta2*, and *APC*, was able to differentiate malignant from nonmalignant cases with 86% sensitivity and 89% specificity [111]. A similar combination of four genes *GSTP1*, *PTGS2*, *RPRM*, and *TIG1* was reported using serum samples [112]. Individually, methylation status of *GSTP1* was most specific (98%) in discriminating CaP and BPH with low sensitive (42–47%). Combination of methylation patterns of four genes provides slightly high specificity (AUC = 0.699) as biomarker [112].

Another class of epigenetic modifications which has been utilized to separate CaP from normal tissue is histone modifications. In a tissue microarray analysis of 113 CaP and 23 non-malignant prostate tissues significant reduction of H3K4me1, H3K9me2, H3K9me3, H3Ac, and H4Ac were observed [113]. Also, levels of H3Ac and H3K9me2 were able to separate CaP and non-malignant prostate tissue with high specificity (>91%) and sensitivity (>78%) [113]. Histone modifications in body fluids have not been studied in detail but one study used ELISA to study global levels of H3K27Me3. This study showed significant decrease in H3K27Me3 in metastatic disease (n = 28) compared to localized disease (n = 33) [114].

### 3.2. Diagnostic and Prognostic miRNA Expression Patterns

Tumor-specific miRNA patterns are emerging as highly attractive biomarkers of cancer risk and progression. Given miRNAs are secreted into body fluids [115] and can be reliably extracted and measured [116], miRNAs offer significant clinical potential as highly sensitive serum-borne prognostic indicators [51,117]. There have been several studies for miRNAs as biomarkers using serum, plasma and urine samples (as reviewed in [118]). Mitchell *et al*. for the first time reported the serum expression of miR-141 was highly elevated in advanced CaP (n = 25) as compare to healthy men (*n* = 25) [51]. Expression of miR-141 was also correlated with serum PSA levels and could detect individuals with advanced PCa with 60% sensitivity at 100% specificity [51]. Expression of miR-21 has been shown to be elevated in hormone-refractory prostate cancer especially in those resistant to docetaxel-based chemotherapy [119]. In another approach Selth *et al*. identified miRNA signatures in TRAMP model and then validated four miRNAs in CaP serum. They identified miR-141, miR-298, and miR-375 to be upregulated in advance CaP [120]. In a candidate approach Yaman Agaoglu *et al*. identified miR-21 (AUC, 88%) and miR-221 (AUC, 83%) as markers to differentiate normal and CaP individuals while miR-141 was able to distinguish localized/local advanced disease (AUC = 0.755) [121]. In a similar candidate approach four miRs (miR-26a, miR-32, miR-195, miR-let7i) were tested in CaP serum samples and showed increased diagnostic accuracy in combination (sensitivity: 78.4%; specificity: 66.7%; AUC: 0.758) [122].

More recently, analysis of 742 miRNAs in plasma-derived circulating microvesicles of 78 prostate cancer patients and 28 normal control individuals identified 12 miRNAs differentially expressed in prostate cancer patients compared to controls [125]. They also confirmed the association of miR-141 and miR-375 with metastatic prostate cancer in a separate cohort of patients. Interestingly, five miRNAs identified in serum samples were also detected in urine samples. miR-107 and miR-574-3p were measured at significantly higher concentrations in the urine of men with prostate cancer compared to controls [123]. MiRNA expression has also been reported to correlate with other clinical parameters. miR-141, miR-200b and miR-375 showed upregulation in CaP and correlate with increasing tumor stage and Gleason score [124]. All together these studies show high potential of miRNAs to be non-invasive biomarkers of CaP. Additionally miRNAs can also help predict disease progression and drug response.

### 3.3. Insights from the Cancer Genome Atlas (TCGA) and ENCODE

**Figure 2:**
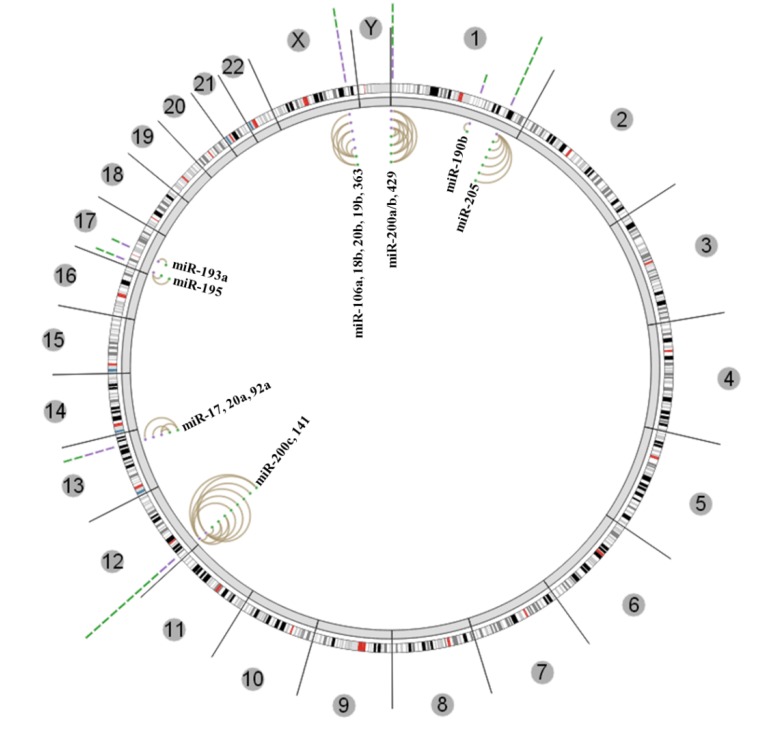
Significant negative correlation between altered miRNA expression and DNA methylation pattern in primary prostate cancer tumors from the TCGA data-set. Circos plot showing miRNAs where there is a significant correlation with CpGs near miRNA gene locations. Outer ring represents all the chromosomes. MiRNA genes are represented by purple lines near their chromosomal locations. Green lines represent CpGs in the region. Lines connecting two dots represent the statistically significant correlation between two features selected, miRNA expression and CpG methylation in this case. (Figures generated from [125] using latest version of data release for Prostate cancer “PRAD-13-March-2013”).

We explored TCGA data for associations between DNA methylation and miRNA expression in CaP tumors. An integrated tool of all the data types generated by TCGA have been developed to understand the systems level interaction between different data features. Statistically significant association can be identified and visualized using Regulome Explorer [125]. Regulome Explorer has been used for integrated analysis in colon, rectal and breast cancers [126,127]. We used Regulome Explorer to identify statistically significant correlations between miRNA expression (RNA-seq) and status of DNA methylation (Illumina 450k methylation arrays). We specifically searched for CpG sites showing negative correlation with miRNA expression on the same chromosome (using “cis” setting in distance filter and p-value cutoff of −log10(p) ≥ 6). This analysis identified a total of 27 CpGs negatively correlated with expression of 17 miRNAs from 5 different chromosomes (Figure 2). Notably corroborating the discussion above, all five miRNAs of miR-200 family (miR-200a/b/429, miR-200c/141) and miR-205 showed strong negative correlation between miRNA expression and CpG methylation [89,90]. Many of miRNAs with important function in CaP did not show significant correlation in this analysis which can be partially because of the allocation of CpGs to miRNAs in Illumina 450K array annotation. This preliminary exploration of TCGA data strengthens the observation that miRNAs are epigenetically silenced in CaP. It will be interesting to interrogate this resource in further detail to identify how other genomic data from TCGA correlates with miRNA expression and DNA methylation.

**Figure 3:**
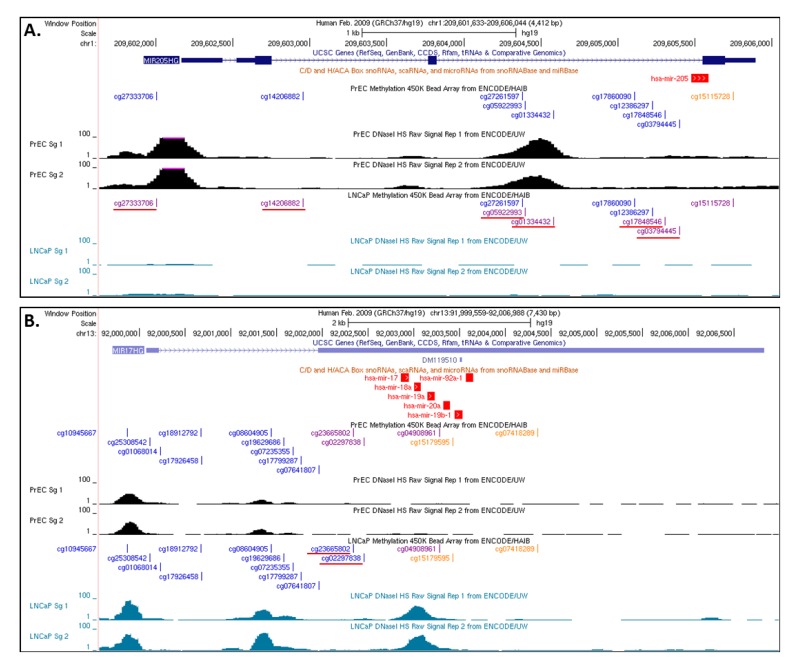
UCSC genome browser tracks showing CpG methylation and DNase I cleavage status around miRNAs. Top panel track shows the genomic location of the miRNA. Middle track shows the CpGs in genomic fragment and color of each CpG represent the beta values (ENCODE description—Orange, beta value equal to or greater than 0.6 was considered fully methylated; Purple, Beta values between 0.2 and 0.6 were considered to be partially methylated; Blue, beta value equal to or less than 0.2 was considered to be fully unmethylated). Bottom panel shows the DNaseI sensitivity measured in PrEC (black) and LNCaP (blue). DNaseI hypersensitive sites shown as peaks suggest accessible chromatin; conversely no DNaseI hypersensitive sites suggest closed chromatin. (**A**) Shows the status of miR-205. Most of the CpGs around miR-205 are partially methylated in LNCaP and un-methylated in PrEC. Supportively, DNaseI sensitivity shows open areas in PrEC cells and closed chromatin in LNCaP and. (**B**) Shows the status of a miR-17-92 cluster. Two CpGs around this cluster are partially methylated in PrEC cells and un-methylated in LNCaP. Supportively, DNaseI sensitivity shows open chromatin in LNCaP and closed in PrEC cells. Differentially methylated CpGs are underlined.

Recently published large scale ENCODE ChIP-seq data for various epigenetic features (e.g., histone marks, chromatin accessibility and DNA methylation) provide a key opportunity to study these associations in various cancer models including CaP [26,128]. Additionally, as an example, we also examined the chromatin accessibility around the miRNAs identified from regulome analysis in LNCaP and PrEC cells from ENCODE. As shown in Figure 3, miRNAs with higher expression in CaP tumors (miR-17, miR-20a and miR-92a) which were associated with low methylation also showed low methylation and open chromatin in LNCaP as compared to PrEC cells. Similarly, miRNA with low expression and high methylation in CaP tumors showed more methylation and closed chromatin in LNCaP (e.g., miR-205).

## 4. Conclusions

The de-regulated cross-talk between epigenetic states and miRNA transcription and action is functionally important in the progression of CaP. Studies utilizing components of epigenetic machinery may offer highly accurate, and accessible, information about CaP diagnosis and prognosis. For example, urine-borne DNA methylation patterns in combination with serum miRNA patterns offer the opportunity to be exploited as highly accurate functional biomarkers. Also, they can be used to understand the underlying epigenetic status to be prognostic of CaP disease progression risks. These components have a strong likelihood to be exploited as integrated non-invasive biomarker of CaP progression and drug resistance.
